# Comparison of ticagrelor with clopidogrel on quality of life in patients with acute coronary syndrome

**DOI:** 10.1186/s12955-021-01875-w

**Published:** 2021-10-16

**Authors:** Hyeyeon Moon, Yoon-Sung Jo, Soo-Jin Kim, Sua Jo, Kyungil Park

**Affiliations:** Regional Cardiovascular Center, Dong-A University Hospital, Division of Cardiology, Department of Internal Medicine, Dong-A University College of Medicine, Daesingongwon 26, Seo-gu, Busan, 49201 Republic of Korea

**Keywords:** Ticagrelor, Clopidogrel, Health-related quality of life, Acute coronary syndrome

## Abstract

**Background:**

Ticagrelor has a Class I recommendation for use following percutaneous coronary intervention (PCI) in acute coronary syndrome (ACS). However, ticagrelor needs to be taken twice a day, as compared to clopidogrel. Its adverse effects, such as dyspnea or bleeding, are known to be more common than with clopidogrel. Dyspnea may tend to be uncomfortable and limit activity. Major bleeding often leads to hospitalization or transfusions, and frequent minor bleeding, which might not result in patients seeking medical care, can make ACS patients feel unhealthy. Thus, these characteristics may affect the health-related quality of life (HQOL).

**Methods:**

In the PLEIO (*comParison of ticagreLor and clopidogrEl on mIcrocirculation in patients with acute cOronary syndrome*) trial, we randomized 120 participants to receive ticagrelor 90 mg twice daily or clopidogrel 75 mg once daily for at least 12 months. We carried out an HQOL assessment with the Short Form 36 Health Survey (SF-36) questionnaire on the day of discharge following PCI, as well as six months later.

**Results:**

At discharge, the HQOL measures were similar in the ticagrelor and clopidogrel groups, both having a physical component summary (PCS) and a mental component summary (MCS) score. A six-month HQOL follow-up assessment showed that there were no differences between the two study groups in either the PCS or the MCS scores. In both groups, the PCS scores significantly increased over six months of treatment (both *p* < 0.01). However, the MCS score did not differ significantly. A baseline MCS score is an independent predictor of better physical and mental health status at six months.

**Conclusions:**

Ticagrelor, as compared to clopidogrel, did not significantly reduce the HQOL during the six months following PCI in patients with ACS.

*Clinical Trial Registration* URL: http://www.clinicaltrials.gov. Unique identifier: NCT02618733.

## Introduction

Antiplatelet agents are the cornerstone drugs for acute coronary syndrome (ACS) in patients who have undergone percutaneous coronary intervention (PCI) [1, 2]. The emerging P2Y12 inhibitor ticagrelor has shown an impressive mortality benefit for ACS patients [3, 4]. According to current clinical practice guidelines, ticagrelor has a Class I recommendation for use following PCI in ACS, with and without ST-segment elevation [1, 2]. However, ticagrelor, which is taken twice daily, could be inconvenient for patients compared to clopidogrel, taken once a day. Side effects, such as dyspnea or bleeding, are known to be more common with ticagrelor than with clopidogrel [3–5]. The Platelet Inhibition and Patient Outcomes (PLATO) study demonstrated that significant increase was seen with ticagrelor therapy, with a 13.8% incidence of dyspnea (versus 7.8% with clopidogrel) with ticagrelor 90 mg twice daily and a 16.1% incidence of major or minor bleedings (versus 14.6% with clopidogrel) [3]. Dyspnea may tend to be uncomfortable and limit activity [5]. Major bleeding often leads to hospitalization or transfusions, and frequent minor bleeding, which might not result in patients seeking medical care, can make ACS patients feel unhealthy [6]. These ticagrelor disadvantages, as compared to clopidogrel, may affect health-related quality of life (HQOL), which is associated with poor adherence to antiplatelet treatment and the eventual prognosis in ACS patients [7–9]. Previous studies have documented the low adherence with ticagrelor [10, 11], which can limit its effectiveness [12]. Therefore, it would be important to assess the HQOL in ACS patients taking ticagrelor. However, previous HQOL studies related to ticagrelor were limited trials that investigated HQOL in a cross-section [13], or they only evaluated bleeding events [14]. Moreover, these studies did not provide an individual evaluation of health status. To date, no clinical trial has been done comparing ticagrelor to clopidogrel on HQOL changes over time. The purpose of this study is to compare the temporal HQOL changes between the ticagrelor and the clopidogrel groups using the 36-Item Short-Form Health Survey (SF-36).

## Methods

### Study design

This was a substudy of the PLEIO (comParison of ticagreLor and clopidogrEl on mIcrocirculation in patients with acute cOronary syndrome) clinical trial, the results of which had been published previously [15, 16]. Briefly, the PLEIO trial was a non-blinded, open-label, and randomized prospective study which was done in a single center. The authors enrolled a total of 120 ACS patients who had been treated with PCI into the PLEIO study. The participants were randomized to receive ticagrelor 90 mg twice daily or clopidogrel 75 mg once daily for at least 12 months.

Patients enrolled in the PLEIO study completed the HQOL assessment questionnaire on the day of discharge and again filled out this questionnaire six months later. We excluded patients from this study if we could not obtain a six-month follow-up HQOL assessment. After the questionnaire had been completed, we conducted an internal review and excluded any incomplete responses [17]. We compared the HQOL in patients randomized to antiplatelet therapy at baseline and at a six-month follow-up. In each group, the temporal change in HQOL between baseline and six months was investigated. The study protocol was approved by the institutional review board. The study was conducted in accordance with the International Conference on Harmonization Guidelines and the tenets of the Declaration of Helsinki. Written informed consent was obtained prior to inclusion in the study. This substudy was preregistered, along with the PLEIO trial [18].

### HQOL assessment

We did an HQOL assessment with the Korean version of the SF-36, v2 questionnaire. This evaluates the HQOL associated with the patient's physical and mental health [19]. A license for the use of the SF-36 v.2 was obtained from OptumInsight Life Sciences, Inc. (license number QM027885). It contains 36 multiple-choice items and had the following eight subscales: physical function, bodily pain, role limitations because of physical functioning, general health perceptions, vitality, mental health, social function, and role limitations due to emotional problems. These eight subscale scores represent values ranging from zero to 100. The physical component summary (PCS) and the mental component summary (MCS) scores were computed from the eight subscales using the special algorithms, which are strictly controlled by a private company [20]. In this study, PCS and MCS scores were used as a measure of HQOL. Higher values indicate a better HQOL [21]. Individual patients were supported by physicians or psychologists unrelated to this study to impart a more suitable interpretation of the questionnaire items and to improve the data quality.

### Adverse events assessment

In the present study, we conducted an in-hospital clinical follow-up of the patients at four weeks and at six months following PCI. We assessed the enrolled patients’ bleeding and antiplatelet agent-related dyspnea at each visit. We categorized each bleeding event according to the Bleeding Academic Research Consortium (BARC) definition [22]. In this study, our diagnosis of antiplatelet agent-related dyspnea was a diagnosis of exclusion. For assessment of antiplatelet-related dyspnea, we conducted patient interviews to assess dyspnea characteristics and to determine whether they had had the identical symptoms before starting antiplatelet therapy. In addition to the patient interviews, we excluded alternative dyspnea causes by physical examination and by other analyses and tests.

### Statistical analysis

The data was summarized as a mean ± standard deviation (SD) for normally distributed continuous variables and percentages for categorical variables. The Shapiro–Wilk test was used to check the data for normal distribution. Comparisons of continuous variables between the ticagrelor and clopidogrel groups were analyzed by using the independent Student t-test or Mann–Whitney U test, depending on normality test of variables. Internal consistency of the SF-36 subscales was evaluated using the Cronbach’s coefficient α. Internal consistency was considered adequate if Cronbach’s coefficient α values were > 0.70 [17].

To examine the antiplatelet therapy-related impact on the HQOL course, we chose the temporal change in PCS or MCS scores per six months as the main analytic parameter. We used a paired-samples t-test to determine whether the SF-36 baseline score had changed over the course of six months following PCI. In this study, we defined a minimally important change (MIC) from the baseline as being more than 3.8 points in the PCS or 4.6 points in the MCS [20]. The MIC was tested with a χ2 or Fisher's exact test. Multivariate logistic regression analyses were performed to identify predictors independently associated with the MIC in HQOL. These independent variables were chosen based on factors associated with HQOL in previous studies [23–32]. As reported in the literature, the following covariates were included in multivariate logistic regression analyses: gender, age, diabetes, hypertension, clinical presentation, cardiac rehabilitation, bleeding events, baseline PCS scores, and baseline MSC scores. We used a criterion of *p* < 0.1 to do univariate analysis to assess the relevance each of these chosen variables to the model. Regression analyses were conducted separately for the dependent MCS and PCS variables. Statistical significance was considered to be at *p* < 0.05. We used the SPSS 16.0 (Statistical package for the Social Sciences, Chicago, Illinois) to do the data analysis.

## Results

### Patient characteristics

**Fig. 1:**
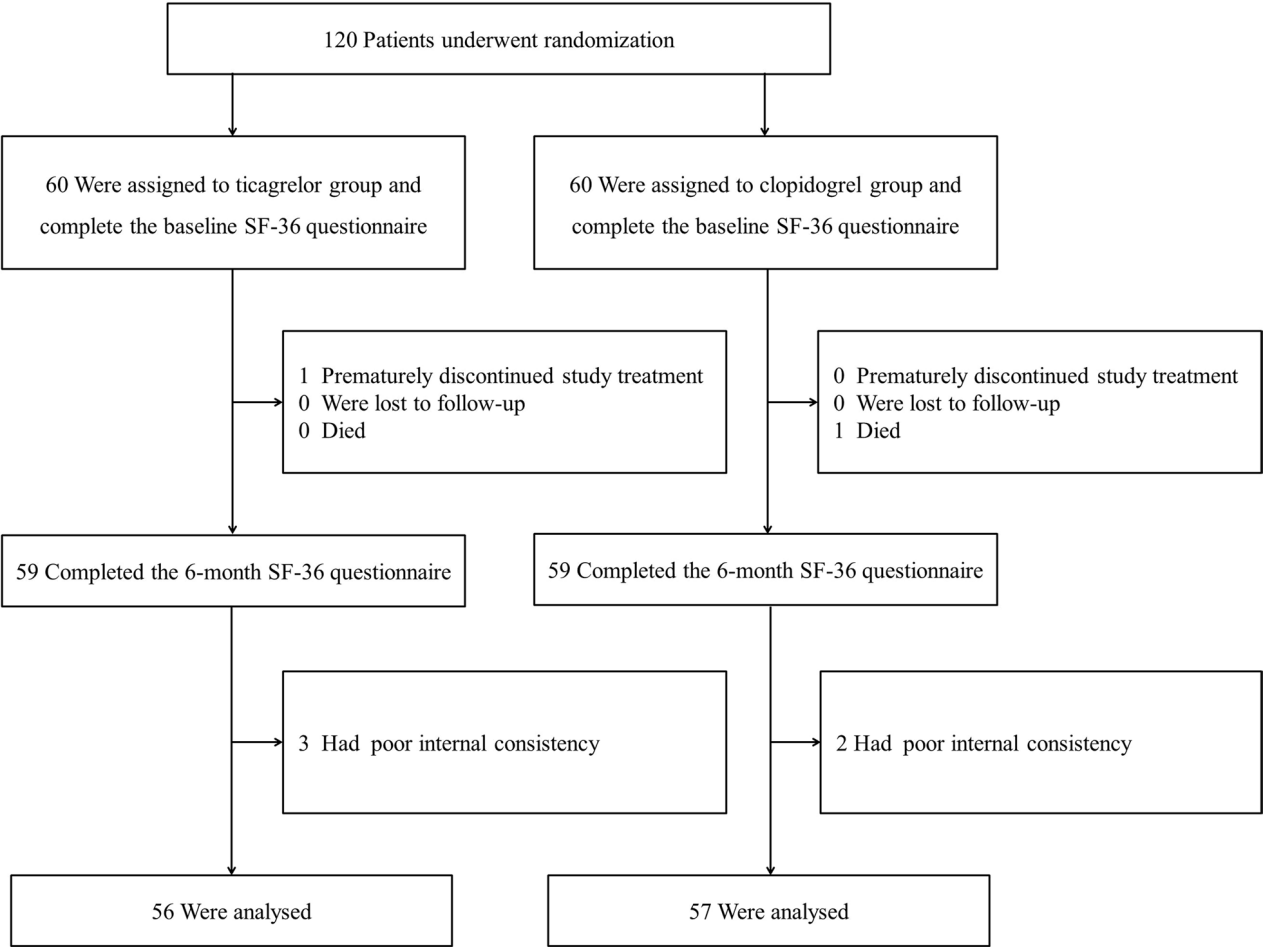
Study flow chart showing the enrollment of patients by using the 36-Item Short-Form Health Survey

Among 120 patients enrolled in the PLEIO study, only one patient (1.7%) in the ticagrelor group stopped the study medication treatment prematurely because this person did not understand how to take it. One patient in the clopidogrel group died of noncardiac causes during the follow-up [15]. One hundred and eighteen patients who had not discontinued the drugs were evaluated for HQOL at discharge and after six months. The SF-36 data of five patients was not available for analysis because of poor internal consistency, and these patients were excluded. Thus, both a complete baseline and six months of data were available for 94.2% (*N* = 113) of the patients in the PLEIO trials (Fig. 1). The final study population consisted of 113 patients, including 56 (49.6%) in the ticagrelor group and 57 (50.4%) in the clopidogrel group. Their characteristics are listed in Table 1. The mean age was 58.9 ± 12.2 years, and 19 (16.8%) of these patients were female. There were 36 (31.9%) patients with STEMI in this population. The baseline characteristics of the two groups were well balanced. Except for ticagrelor and clopidogrel, there was no difference between the two groups in the number or type of medications taken. We compared a severity score between the two groups using the Global Registry of Acute Coronary Events (GRACE) score which is widely used as an acute risk stratification tool in the evaluation of prognosis in patients with ACS [33]. According to GRACE score, there was no statistical difference in disease severity between the two groups (*p* = 0.46).

**Table 1:** Baseline patient characteristics in the health-related quality of life substudy population

Characteristics	Ticargrelor (N = 56)	Clopidogrel (N = 57)
Female sex, N (%)	8 (14.3%)	11 (19.3%)
Age, years	57.9 ± 12.4	58.1 ± 11.3
Body mass index, kg/m^2^	25.2 ± 3.8	24.9 ± 2.9
Risk factors		
Diabetes mellitus, N (%)	21 (37.5%)	20 (35.1%)
Hypertension, N (%)	24 (42.8%)	25 (43.9%)
Dyslipidemia, N (%)	17 (30.4%)	16 (28.1%)
Current smoker, N (%)	33 (58.9%)	25 (43.9%)
Ejection fraction, %	52.1 ± 9.0	52.2 ± 9.7
Clinical presentation		
Unstable angina, N (%)	13 (23.2%)	16 (28.1%)
NSTEMI, N (%)	26 (46.4%)	22 (38.6%)
STEMI, N (%)	17 (30.4%)	19 (33.3%)
Discharge medication		
Beta-blocker, N (%)	54 (96.4%)	52 (91.2%)
ACE inhibitor or ARB, N (%)	49 (87.5%)	48 (84.2%)
Statin, N (%)	56 (100.0%)	57 (100.0%)
Aspirin, N (%)	56 (100.0%)	57 (100.0%)
Cardiac rehabilitation, N (%)	51 (91.1%)	50 (89.3%)
Number of vessels diseased		
1 vessel disease, N (%)	38 (67.8%)	36 (63.1%)
2 vessel disease, N (%)	15 (26.8%)	18 (31.6%)
3 vessel disease, N (%)	3 (5.4%)	3 (5.3%)
GRACE score	109 ± 21	110 ± 18
Laboratory findings		
Serum hemoglobin, g/dl	14.4 ± 1.8	14.1 ± 1.3
Platelet count, × 10^9^/L	24.54 ± 5.5	23.70 ± 7.4
Serum creatinine, mg/dL	1.0 ± 0.3	1.0 ± 0.2
Cardiac troponin I, ng/mL	48.0 ± 60.7	49.4 ± 68.8
Brain natriuretic peptide, pg/mL	60.2 ± 100.1	73.1 ± 93.6
LDL cholesterol, mg/dL	120.9 ± 36.2	119.0 ± 33.8
HDL cholesterol, mg/dL	42.9 ± 9.8	44.9 ± 12.5
Total cholesterol, mg/dL	187.6 ± 47.4	194.6 ± 47.7
Triglycerides, mg/dL	174.4 ± 130.3	170.1 ± 104.4

Data were expressed as means ± SD or n (%)

ACE, angiotensin-converting-enzyme; ARB, angiotensin-receptor blocker; BNP, B-type natriuretic peptide; GRACE, Global Registry of Acute Coronary Events; HDL, high-density lipoprotein; LDL, low-density lipoprotein; NSTEMI, Non–ST-segment–elevation myocardial infarction; STEMI, ST-segment–elevation myocardial infarction

### Comparison of HQOL between the ticagrelor and clopidogrel groups

The baseline and six-month follow-ups of the HQOL using the SF-36 were similar between the ticagrelor and clopidogrel groups (Table 2). At baseline, both the PCS (51.2 ± 6.7 versus 50.8 ± 7.2, *p* = 0.78) and MCS (50.7 ± 6.7 versus 50.3 ± 7.5, *p* = 0.80) scores were similar between the two groups. Six-month HQOL follow-up data showed that PCS (55.6 ± 3.8 versus 55.6 ± 3.9, *p* = 0.97) and MCS (51.8 ± 3.9 versus 51.4 ± 4.2, *p* = 0.65) scores did not differ significantly between the two groups. The scores were similar in both groups at baseline and at six-month follow-up for all subscales: physical function, bodily pain, role limitations due to physical functioning, general health perceptions, vitality, mental health, social function, and role limitations owing to emotional problems (Table 2).

**Table 2:** Health-related quality of life between the ticagrelor and clopidogrel groups

Study outcomes	Ticargrelor (N = 56)	Clopidogrel (N = 57)	p value
Baseline measures			
Physical component summary	51.2 ± 6.7	50.8 ± 7.2	0.78
Physical functioning	84.4 ± 17.2	83.3 ± 16.6	0.71
Role physical	87.3 ± 19.6	86.2 ± 19.5	0.73
Bodily pain	74.5 ± 24.6	73.2 ± 23.2	0.61
General health	57.8 ± 17.5	58.8 ± 17.5	0.53
Mental component summary	50.7 ± 6.7	50.3 ± 7.5	0.80
Vitality	59.4 ± 18.2	58.8 ± 18.8	0.85
Social functioning	91.4 ± 16.8	89.4 ± 18.6	0.69
Role emotional	88.6 ± 19.4	89.4 ± 18.6	0.81
Mental health	70.5 ± 14.9	70.7 ± 15.9	0.95
6-month measures			
Physical component summary	55.6 ± 3.8	55.6 ± 3.9	0.97
Physical functioning	91.2 ± 11.4	90.6 ± 11.1	0.77
Role physical	95.4 ± 11.7	96.7 ± 8.4	0.48
Bodily pain	93.8 ± 13.2	94.4 ± 8.0	0.72
General health	62.6 ± 13.1	64.5 ± 13.4	0.16
Mental component summary	51.8 ± 3.9	51.4 ± 4.2	0.65
Vitality	63.2 ± 12.8	62.4 ± 13.1	0.75
Social functioning	99.0 ± 4.7	97.5 ± 12.7	0.18
Role emotional	97.2 ± 7.7	96.6 ± 9.9	0.76
Mental health	72.1 ± 8.9	72.1 ± 10.8	0.99

Data was expressed as means ± SD

### Temporal changes in HQOL during six-month follow-up

**Fig. 2:**
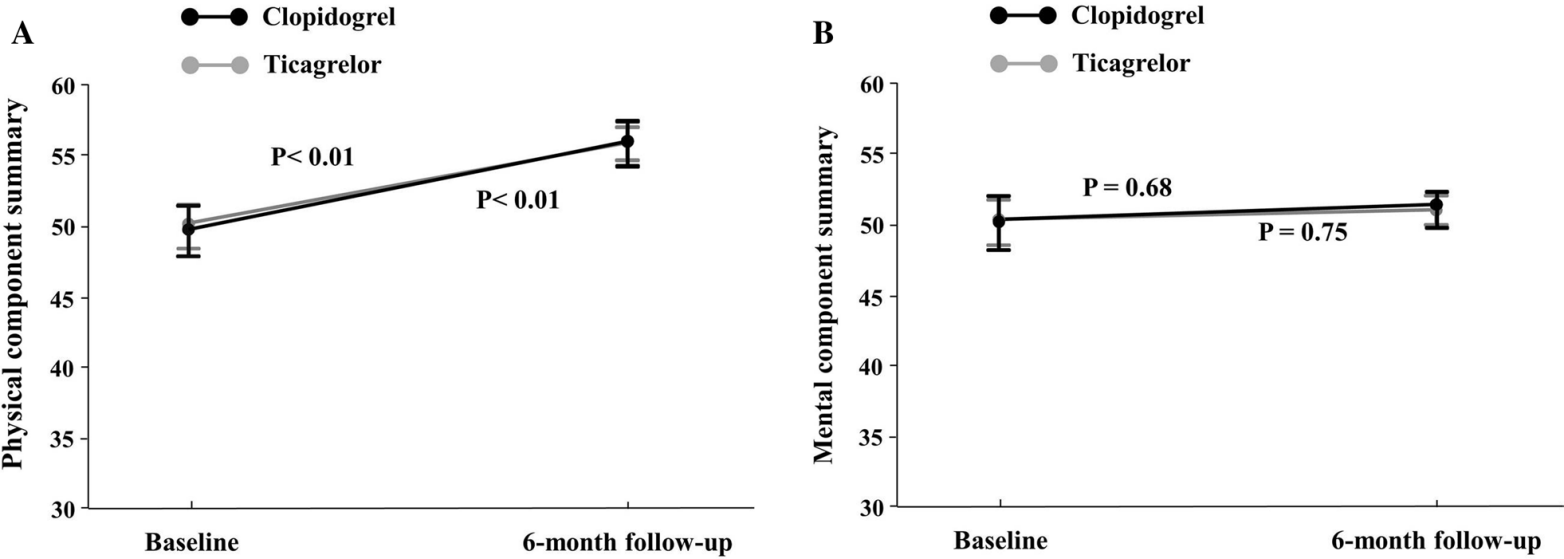
Mean scores over times in PCS and MCS of the SF-36 in ticagrelor and clopidogrel groups. Shown are mean (± SE) measure levels of PCS (Panel **A**), are mean (± SE) measure levels of MCS (Panel **B**) from baseline to 6 months in the two study groups. *MCS* mental component summary, *PCS* physical component summary

Temporal changes over a six-month period showed an increase in PCS from 51.2 ± 6.7 to 55.6 ± 3.8 in the ticagrelor group (*p* < 0.01), and from 50.8 ± 7.2 to 55.6 ± 3.9 in the clopidogrel group (*p* < 0.01). Over a six-month period, baseline scores improved in both groups for all PCS subscales (*p* < 0.01 for all comparisons). However, for six months, the MCS did not change significantly in either group. Figure 2 details the mean PCS and MCS values from baseline to six months in both groups. The MIC in the PCS and MCS over time was reported to be 52.6% and 19.5%, respectively. There was no significant difference in MIC occurrences between the ticagrelor or clopidogrel groups.

In the multivariate regression model, we included the following baseline variables: female sex, age, diabetes, hypertension, clinical presentation, cardiac rehabilitation, any BARC bleeding, ticagrelor use, baseline PCS, and baseline MCS. In univariate analysis, female sex (OR: 0.75; 95% CI: 0.40 to 0.90; *p* = 0.01), age (OR: 0.82; 95% CI: 0.35 to 0.98; *p* = 0.04), diabetes (OR: 0.62; 95% CI: 0.37 to 1.45; *p* = 0.08), hypertension (OR: 0.79; 95% CI: 0.47 to 1.22; *p* = 0.09), clinical presentation (OR: 0.88; 95% CI: 0.67 to 2.14; *p* = 0.09), baseline MCS score (OR: 1.98; 95% CI: 1.47 to 3.23; *p* < 0.01), cardiac rehabilitation (OR: 1.45; 95% CI: 1.29 to 1.88; *p* = 0.03) and any BARC bleeding (OR: 0.76; 95% CI: 0.26 to 0.92; *p* = 0.02), were related to the MIC of PCS and MCS. Table 3 shows the multivariate factors associated with the MIC in HQOL. Under a multivariate logistic regression model, factors significantly associated with the MIC in PCS were baseline PCS (OR: 1.98; 95% CI: 1.35–2.93; *p* = 0.03) and baseline MCS (OR: 1.49; 95% CI: 1.22–2.45; *p* = 0.04). However, ticagrelor use was not associated with the MIC in PCS or MCS. The strongest predictors for the MIC in MCS was the baseline MCS score (OR: 2.55; 95% CI: 1.11–3.80; *p* = 0.02).

**Table 3:** Predictors of the minimal important change in health-related quality of life at 6 months after PCI

Variables	Multivariate logistic regression		
	OR	95% CI	p value
*PCS*			
Female	0.93	0.67–1.44	0.10
Age	0.82	0.35–2.08	0.23
Diabetes	0.97	0.63–1.82	0.77
Hypertension	0.92	0.55–2.96	0.86
Clinical presentation	1.78	0.42–3.20	0.83
Cardiac rehabilitation	1.79	0.98–2.31	0.07
Any BARC bleeding	0.89	0.62–1.46	0.41
Ticagrelor use	1.21	0.97–2.07	0.89
Baseline PCS score	1.98	1.35–2.93	0.03
Baseline MCS score	1.49	1.22–2.45	0.04
*MCS*			
Female	0.82	0.40–1.91	0.09
Age	0.80	0.28–1.02	0.07
Diabetes	0.74	0.46–1.89	0.16
Hypertension	0.81	0.44–2.09	0.23
Clinical presentation	0.77	0.57–2.42	0.21
Cardiac rehabilitation	1.99	1.04–2.51	0.08
Any BARC bleeding	0.65	0.36–1.74	0.11
Ticagrelor use	1.12	0.72–1.97	0.86
Baseline PCS score	2.18	0.98–3.36	0.06
Baseline MCS score	2.55	1.11–3.80	0.02

BARC, Bleeding Academic Research Consortium; MCS, mental component summary; PCS, physical component summary

### Adverse events

Bleeding events were published [15]. In this substudy, BARC bleedings occurred in 9 (8.0%) of 113 ACS patients over a six-month follow-up period. BARC-1 bleeding occurred in six (10.7%) patients in the ticagrelor group and in one patient (1.7%) in the clopidogrel group. BARC-3a bleeding occurred in two (3.5%) patients in the clopidogrel group. There was no significant difference between the ticagrelor (*N* = 6, 10.7%) and the clopidogrel groups (*N* = 3, 2.7%) with respect to bleeding events (*p* = 0.21). We did not observe bleeding lasting more than one day in any study group that required interruption of the study drug [15, 34]. Following clinical risk adjustment, no BARC bleeding correlated with baseline changes in the SF-36 component summary scores. At a six-month follow-up, a total of 12 patients (10.6%) had dyspnea that the investigator judged to be related to antiplatelet agents, and 10 of them occurred in the ticagrelor group. Antiplatelet agent-related dyspnea was more frequently reported in the ticagrelor group as compared to the clopidogrel group (17.8% of patients versus 3.5%, *p* = 0.02). However, neither group discontinued the study medication nor limited exercise because of dyspnea. There was no significant correlation between antiplatelet agent-related dyspnea and baseline changes in the SF-36 summary component scores.

## Discussion

Clopidogrel or ticagrelor is the recommended antiplatelet agent for recurrent ischemic events, including stent thrombosis, after ACS. Ticagrelor yields greater inhibition of platelet aggregation than clopidogrel and was shown to reduce the risk of ischemic events and death compared with clopidogrel in the PLATO study. It is therefore recommended as a first-line agent over clopidogrel in ACS [1, 2]. The most frequent adverse events of ticagrelor are dyspnea and bleeding [3–5]. Ticagrelor-related dyspnea is generally mild and transit [5]. Occasionally, dyspnea tends to be uncomfortable and may limit activity, potentially affecting patients’ HQOL. Bleeding is the major adverse event of antiplatelet therapy and clinically important [6, 35]. Patients with ACS who experience a major bleed have significantly longer hospital stays, higher readmission rates, and increased mortality than those without a major bleed [6]. Besides, it can cause patients to discontinue antiplatelet therapy, thereby increasing their risk of thrombosis [36]. Frequent minor bleeding, which might not result in patients seeking medical care, can make ACS patients feel unhealthy. These events eventually can have impact on HQOL.

This substudy compared HQOL between ticagrelor and clopidogrel at baseline and at a six-month follow-up of ACS patients. The main findings are as follows: First, the HQOL did not differ between the ticagrelor or clopidogrel groups at baseline or at a six-month follow-up. Second, there was a significant change in PCS but not in the MCS over six months. Third, the baseline MCS score is the most important predictor of both physical and mental HQOL improvement.

In the present study, even though more patients were reported with antiplatelet agent-related dyspnea and bleeding in the ticagrelor group as compared to the clopidogrel group, there was no difference in HQOL between the ticagrelor and clopidogrel groups over a six-month period. These results seem to be caused by several reasons. First, ticagrelor-related dyspnea and bleeding did not appear to be severe enough to affect the HQOL in this study population. Our data suggested that bleeding and dyspnea were not correlated with baseline changes in the SF-36 component summary scores. Second, the follow-up period of 6 months in the study was relatively short to compare the adverse events of antiplatelet therapy. Because of this short period, there was no significant difference in bleeding frequency between the two groups, and as a result, HQOL may have been less affected. Third, these results seem to be caused by effective patient education. In the present study, we assessed the antiplatelet agent-related dyspnea and bleeding at each visit following PCI, and educated the adverse events. This can decrease patients’ concerns about the antiplatelet agents and improve their HQOL [37, 38]. Polypharmacy is associated with poor health outcomes, including medication nonadherence, adverse drug effects, and worse quality of life [39, 40]. Previous studies have reported that reducing dose frequency may offer benefits for the patient in terms of HQOL [41]. Our trial, however, showed that the frequency of dosing twice a day with ticagrelor did not affect HQOL as compared to clopidogrel administered once a day. This substudy also analyzed the association between the number of medications taken and the improvement in HQOL, but no correlation was found. It seems that a slightly increased number of pills ingested per day may have little effect on HQOL.

Poor mental health is a risk factor for morbidity and mortality in ACS patients [42, 43]. As a result, current guidelines recommend screening and appropriate treatment of mental disorders, such as depression and anxiety, in coronary artery disease [1, 2, 44, 45]. Physical and mental health are fundamentally linked and influence each other [46]. Better physical HQOL can affect mental HQOL and vice versa. Many studies have reported that physical health improves after PCI in ACS patients [27, 47, 48]. However, data on mental health improvement is inconsistent [48–52]. Our study showed that in ACS patients, physical HQOL is improved, but during follow-up, the mental component of the HQOL scores is maintained. The present study also revealed that the baseline MCS score is an independent predictor of HQOL improvement in ACS patients. These findings from our research may suggest that mental status has a very strong impact on the patient’s HQOL, notwithstanding positive effects of physical health improvement following PCI in ACS patients [10, 47, 48]. Thus, in addition to the aspect of managing risk factors for ACS patients, assessment and care of mental health may still be important in improving the QOL of ACS patients.

### Study limitation

The present study is, to the best of our knowledge, the first to evaluate the long-term effects of ticagrelor on HQOL, based on the SF-36. However, this study has some limitations. First, this is an underpowered study as the original purpose of the PLEIO study was not for HQOL estimation. So, we did not evaluate depression, anxiety severity, social or economic factors that may affect HQOL. Second, the sample size was relatively small and was composed of a heterogeneous population. Thus, these results need to be corroborated in a larger homogenous population.

## Conclusion

Ticagrelor did not significantly reduce HQOL for six months following PCI in patients with ACS as compared to clopidogrel. Our study suggests that continuation of ticagrelor therapy, even though there are concerns about the risk of adverse events and polypharmacy, is tolerable without loss of HQOL. Following PCI, physical related HQOL improved, but mental related HQOL remained unchanged in ACS patients. To enhance the HQOL of these patients, mental health care is needed from the start of ACS.

## Data Availability

The data for the research presented in the publication may be available from the corresponding author on reasonable request.
